# Supplementary material for: Lactiplantibacillus plantarum 1008 Promotes Reproductive Function and Cognitive Activity in Aged Male Mice with High-Fat-Diet-Induced Obesity by Altering Metabolic Parameters and Alleviating Testicular Oxidative Damage, Inflammation and Apoptosis
Source: Antioxidants (Basel). 2024 Dec 9;13(12):1498. doi: 10.3390/antiox13121498 (PMC11673844; doi:10.3390/antiox13121498)
Supplement: Supplementary file 1 [file antioxidants-13-01498-s001.zip › antioxidants-3283766-supplementary.pdf]

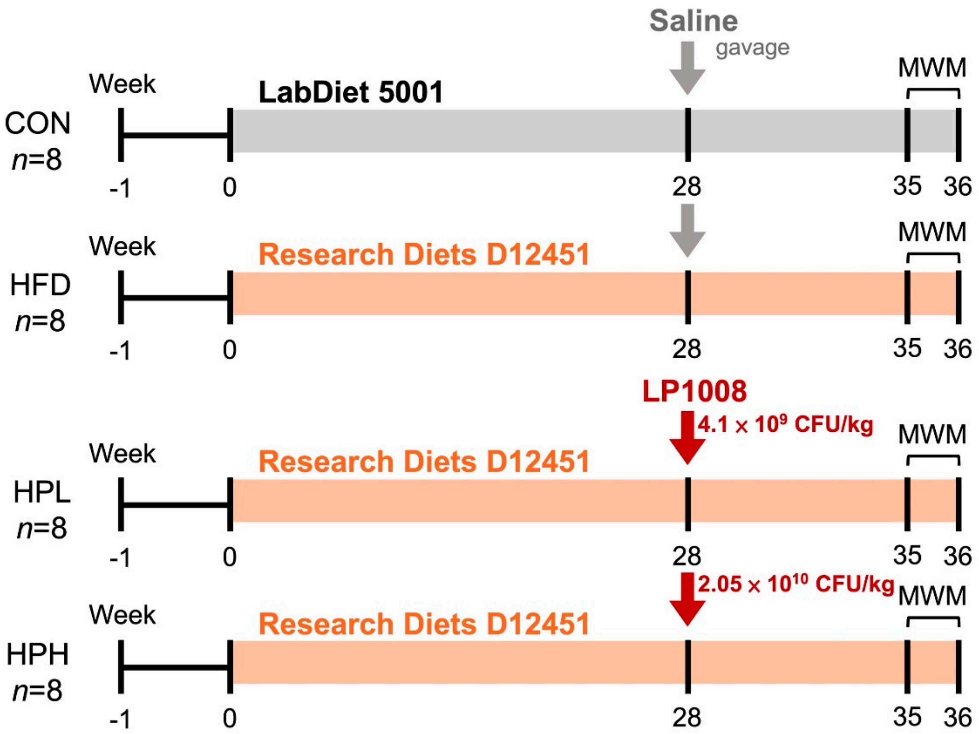

Figure S1. A schematic illustration for the whole experimental flow.

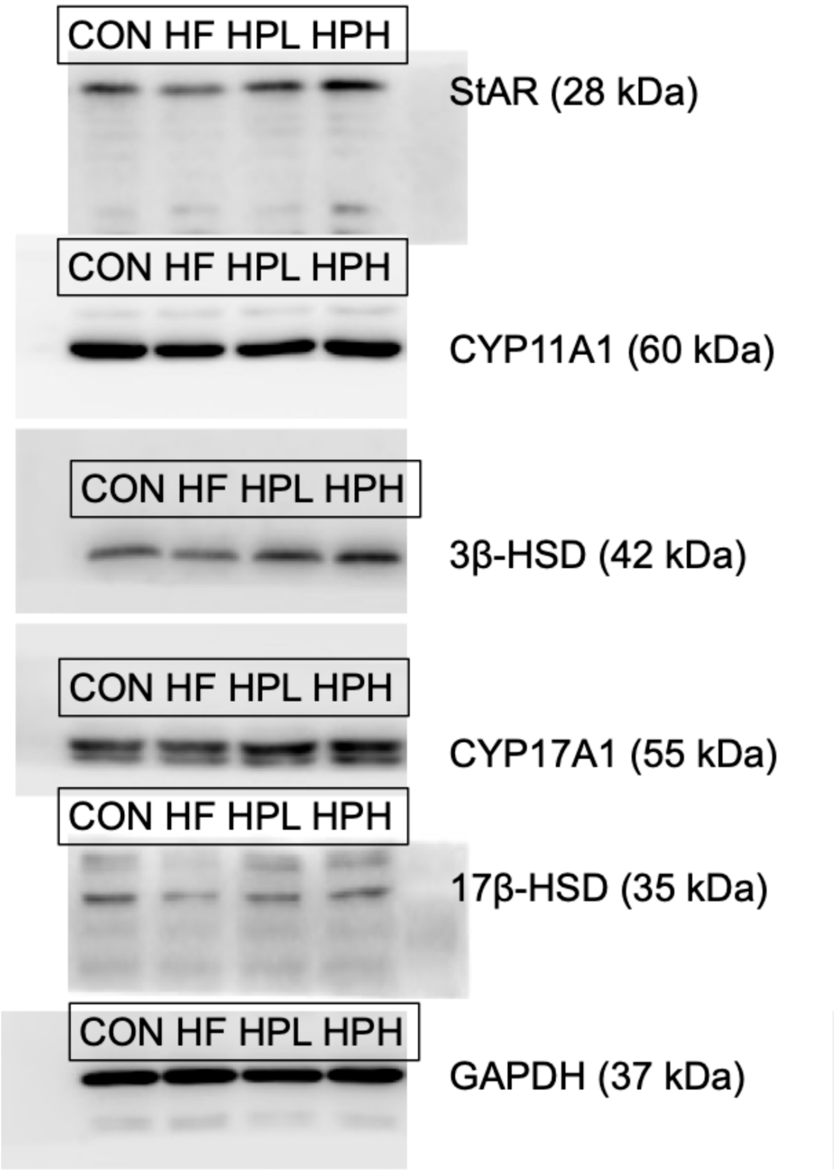

Figure S2. Whole Western blots of testosterone biosynthesis for StAR (28 kDa), CYP17A1 (60 kDa), 3β-HSD (42 kDa), CYP11A1 (55 kDa), 17β-HSD (35 kDa) and GAPDH (37 kDa) in aged and obese mice for Figure 5.

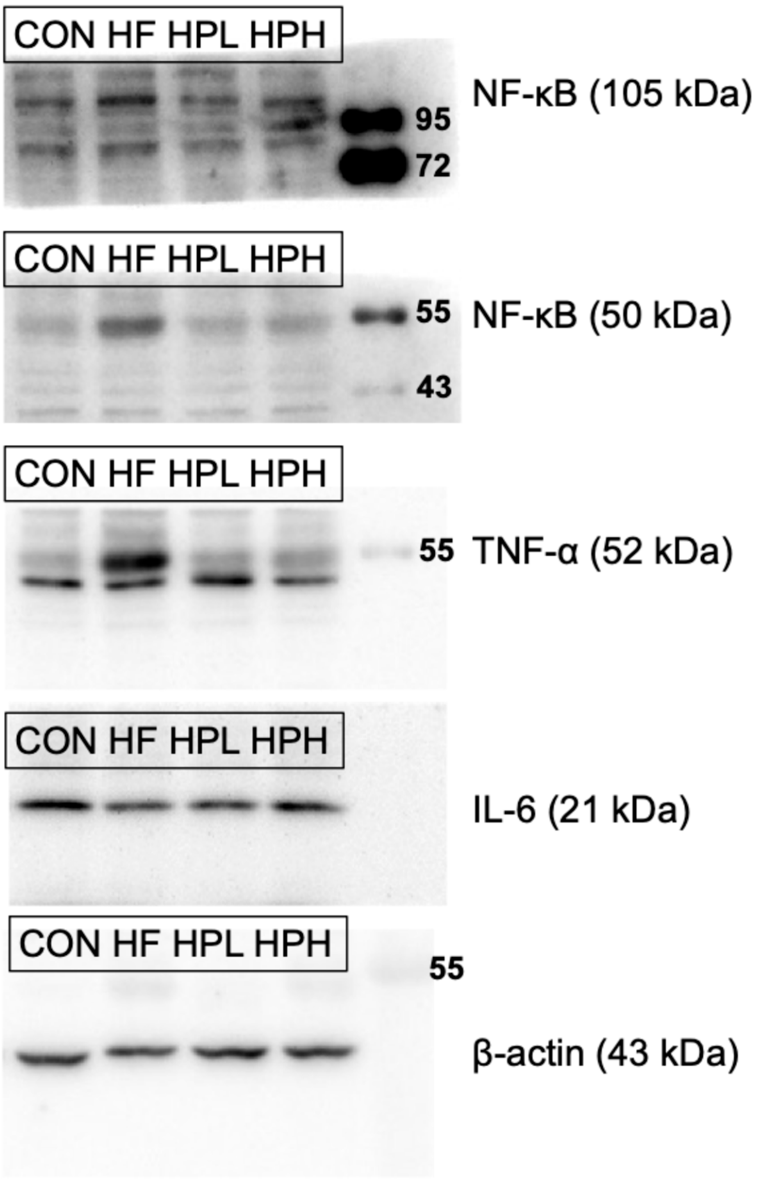

Figure S3. Whole Western blots of inflammation for NF-κB (105 and 50 kDa), TNF-α (52 kDa), IL-6 (21 kDa) and β-actin (43 kDa) in aged and obese mice for Figure 10.

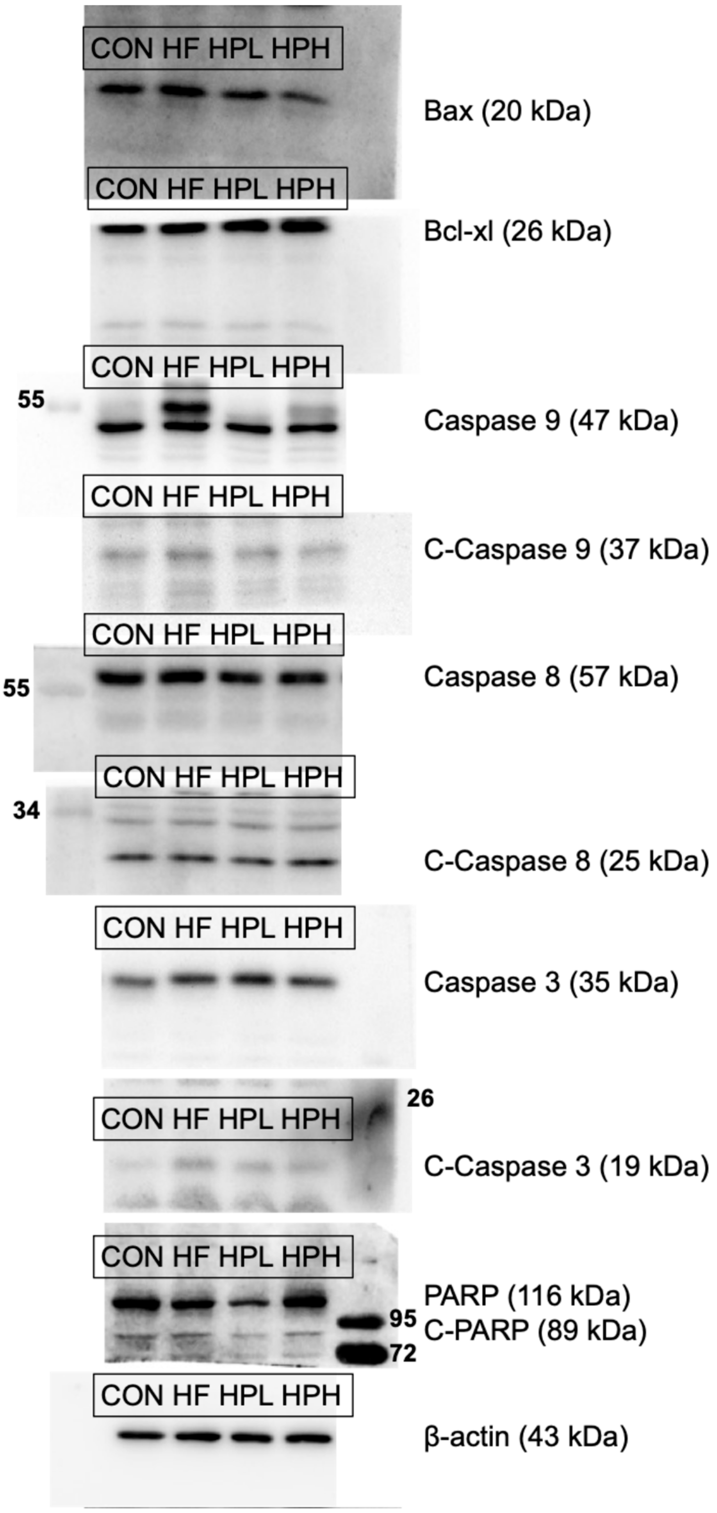

Figure S4. Whole Western blots of apoptosis for PARP (116 kDa), C-PARP (89 kDa), β-actin (43 kDa), Caspase 8 (58 kDa), C-Caspase 8 (25 kDa), Caspase 9 (47 kDa), C-Caspase 9 (37 kDa), Caspase 3 (35 kDa), C-Caspase 3 (19 kDa), Bcl-xl (26 kDa), Bax (20 kDa) and C-Caspase 3 (19 kDa) in aged and obese mice for Figure 11.
